# Sarcopenia in Peritoneal Dialysis: Prevalence, Pathophysiology, and Management Strategies

**DOI:** 10.1016/j.xkme.2025.100989

**Published:** 2025-03-07

**Authors:** Xiaohua Hu, Bibo Wu, Yang Yang, Liming Zhang, Cheng Xue

**Affiliations:** 1 Department of Nephrology, Zhabei Central Hospital of Jing’an District, Shanghai, China; 2 Department of Nephrology, 981th Hospital of PLA, Chengde, Hebei province, China; 3 Department of Nephrology, Shanghai Changzheng Hospital, Second Affiliated Hospital of Naval Medical University (Second Military Medical University), Shanghai, China

**Keywords:** Sarcopenia, peritoneal dialysis, protein-energy wasting, muscle mass, dialysis, treatment

## Abstract

Sarcopenia, defined as the loss of skeletal muscle mass, strength, and function, is a significant complication in patients with chronic kidney disease, particularly those undergoing peritoneal dialysis (PD). This review explores the prevalence, pathophysiology, diagnostic challenges, and management strategies of sarcopenia in the PD population. The multifactorial etiology of sarcopenia in PD, including protein-energy wasting, chronic inflammation, insulin resistance, and hormonal imbalances, underscores the complexity of its management. The prevalence of sarcopenia in patients treated with PD is influenced by age, duration of dialysis, and comorbid conditions, presenting a considerable variation across studies due to differing diagnostic criteria. Diagnostic challenges arise from fluid overload and the PD process, affecting the accuracy of muscle mass measurements. Intervention strategies focusing on nutritional supplementation and physical exercise have shown promise; however, the need for PD-specific diagnostic criteria and treatment protocols remains. This review highlights the critical effect of sarcopenia on functional status and survival in patients treated with PD, emphasizing the importance of addressing this condition to improve patient outcomes. Future directions call for comprehensive, longitudinal studies to better understand sarcopenia’s progression in patients treated with PD and the development of tailored interventions.

Sarcopenia, a degenerative syndrome characterized by reduced skeletal muscle mass, strength, and function, is a prevalent complication in patients with chronic kidney disease (CKD), especially in those with end-stage kidney disease undergoing kidney replacement therapy.^1^ This condition has been identified as a critical predictor of falls, fractures, mobility disorders, dependency, low quality of life, and mortality.^2^^,^^3^ Traditionally, age-related sarcopenia is categorized as primary, whereas secondary sarcopenia, often disease-related, is more common among individuals with kidney failure.^4^ Factors such as the accumulation of uremic toxins, metabolic acidosis, malnutrition, loss of amino acids during dialysis treatment, and a chronic low-grade inflammatory state contribute to the development of kidney failure-related sarcopenia.^5^^,^^6^ These factors lead to a negative nitrogen balance due to increased protein breakdown and decreased protein synthesis. Moreover, the physical inactivity commonly observed in dialysis patients exacerbates muscle loss.^7^

Numerous studies, including patients treated with dialysis and kidney transplant recipients, have been published since the first Sarcopenia Consensus from the European Working Group for Sarcopenia for Older People (EWGSOP) was published.^8^ Peritoneal dialysis (PD) is a modality among kidney replacement therapies. Among the various complications in patients undergoing PD, sarcopenia is an important condition associated with high rates of disability, mortality, and morbidities.^9^ For patients treated with PD, sarcopenia’s impact is profound, affecting their mobility, independence, and overall health outcomes. The condition in these patients is often exacerbated by the catabolic state induced by kidney disease, the dialysis process itself, and the associated comorbid conditions. These factors contribute to the complex interplay of pathophysiological mechanisms leading to muscle wasting and weakness, necessitating a comprehensive approach to diagnosis that considers both muscle mass and function.

This review aims to delve into the conceptual definition of sarcopenia, its etiology, prevalence, predictive markers, association with clinical outcomes, and interventions targeting sarcopenia in patients treated with PD, incorporating the latest research findings to provide a comprehensive overview of this significant complication.

### Definition of Sarcopenia

In 1989, Irwin Rosenberg first proposed the concept of sarcopenia. It has been defined as a clinical syndrome mainly characterized by a decline in skeletal muscle mass, strength, and function.^1^ Secondary sarcopenia is mostly caused by endocrine and metabolic disorders or advanced organ failure, such as heart failure, kidney failure, diabetes, liver cirrhosis, malignant tumors, etc. Among them, uremic sarcopenia caused by kidney failure is the most common.^4^ EWGSOP and the Asian Working Group for Sarcopenia (AWGS) have provided criteria that emphasize the importance of both muscle mass and function (strength or performance) in the diagnosis of sarcopenia. These criteria reflect the multifactorial etiology of sarcopenia, highlighting the contributions of not only chronic illness and inflammation but also nutritional deficits and physical inactivity, which are particularly relevant in patients treated with PD.

In PD, the definition extends beyond the loss of muscle mass to include reductions in muscle function and performance, which are critical for diagnosing and managing the condition effectively. The accurate assessment of sarcopenia in patients treated with PD involves a combination of objective measurements of muscle mass, such as bioelectrical impedance analysis (BIA) or dual-energy X-ray absorptiometry, and functional assessments, such as handgrip strength and physical performance tests. These diagnostic tools enable health care providers to identify sarcopenia at its early stages, allowing for timely interventions aimed at mitigating its impact on the health and quality of life of patients treated with PD.

By understanding and applying these updated definitions and diagnostic criteria, clinicians can better identify sarcopenia in patients treated with PD, leading to improved management strategies that address both the loss of muscle mass and the decline in muscle function, ultimately enhancing patient care and outcomes.

### Prevalence of Sarcopenia

The prevalence of sarcopenia in patients undergoing dialysis exhibits a broad range from 1.5%-68%.^7^ This variability stems from the diverse protocols used across studies to assess patients’ muscle status. In a study by Kamijo et al,^10^ among 119 patients treated with PD, 8.4% were identified with comorbid sarcopenia. Abro et al,^11^ through the evaluation of muscle strength and mass in 155 patients treated with PD, determined that sarcopenia prevalence fluctuated between 11.0% and 15.5%. A meta-analysis highlighted the highest prevalence at 36.9% when employing the AWGS 2019 criteria, with the EWGSOP 2019 criteria indicating a prevalence of 24.1%.^7^ In terms of dialysis modality, sarcopenia was found to be significantly more prevalent in the population treated with hemodialysis (HD) (26.8%) when compared with the population treated with PD (17.5%).^7^ Peritoneal dialysis offers some advantages over HD in preserving muscle mass and function, which might explain the lower prevalence of sarcopenia among patients treated with PD. Younger patients with kidney failure, who are generally in better physical condition, often opt for PD over HD. In addition, patients treated with PD tend to maintain better residual renal function, experience fewer complications, and enjoy improved cognitive function and quality of life than those on HD.^12^ Hung et al^13^ observed a prevalence rate of 2.2%-31.3% in female and 25.1%-75.6% in male patients treated with PD, noting that the higher prevalence observed in males was not directly associated with dialysis treatment.

### Mechanisms Linking PD to Sarcopenia

The development of sarcopenia in patients treated with PD involves a complex interplay of mechanisms that contribute to muscle wasting and decreased muscle function. The factors inherent in sarcopenia and the causes unique to PD jointly aggravate the progression of sarcopenia. Most research reports show that aging, decreased appetite, insufficient nutritional intake, vitamin D deficiency, reduced sex hormones, increased inflammatory cytokines, and overexpression of angiotensin II are the main factors in sarcopenia in early-stage CKD; uremic toxins, metabolic acidosis, lack of exercise, insulin resistance, and reduced dietary intake, nutrient losses into the dialysate, and comorbid conditions are mainly related to sarcopenia in patients treated with dialysis. Altogether, the conditions that patients with CKD, especially those treated with dialysis, are exposed to will result in a negative protein balance that can result in muscle loss, weakness (low muscle strength), low physical performance, disability, and frailty (Fig 1). In patients treated with PD, muscle mass loss is driven by chronic inflammation, metabolic acidosis, and protein-energy wasting, which accelerate muscle protein degradation and impair synthesis. Reduced muscle strength is caused by uremic toxins, oxidative stress, and prolonged physical inactivity, leading to muscle atrophy and neuromuscular dysfunction. Physical performance declines due to muscle wasting, fatigue, and diminished exercise capacity, further exacerbated by anemia, cardiovascular complications, and a sedentary lifestyle. These factors collectively contribute to the progression of sarcopenia, significantly impacting quality of life and prognosis in patients treated with PD. Key factors included above are shown in Fig 2 and listed as follows:

**Figure 1:**
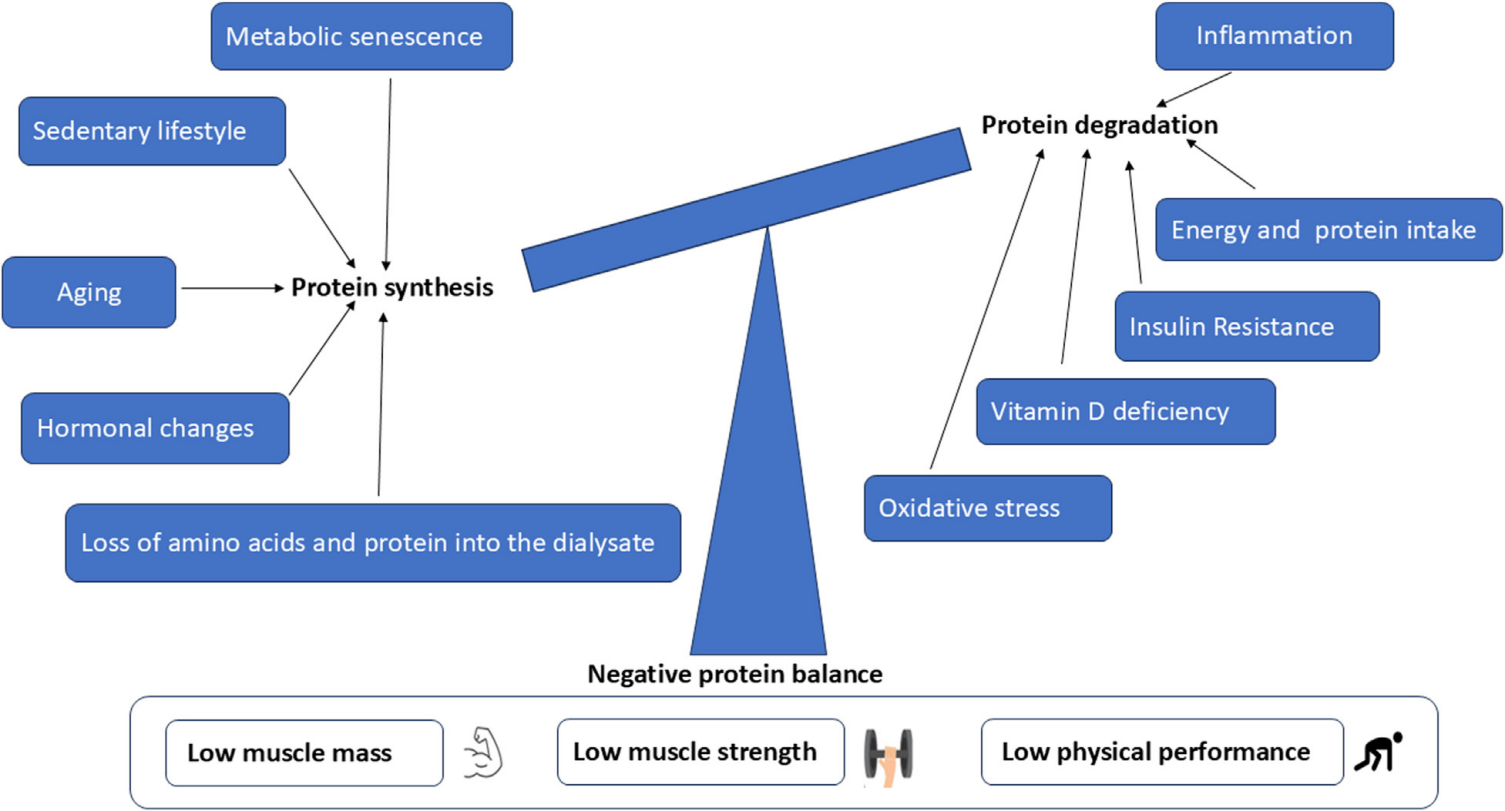
The pathophysiology of sarcopenia.

**Figure 2:**
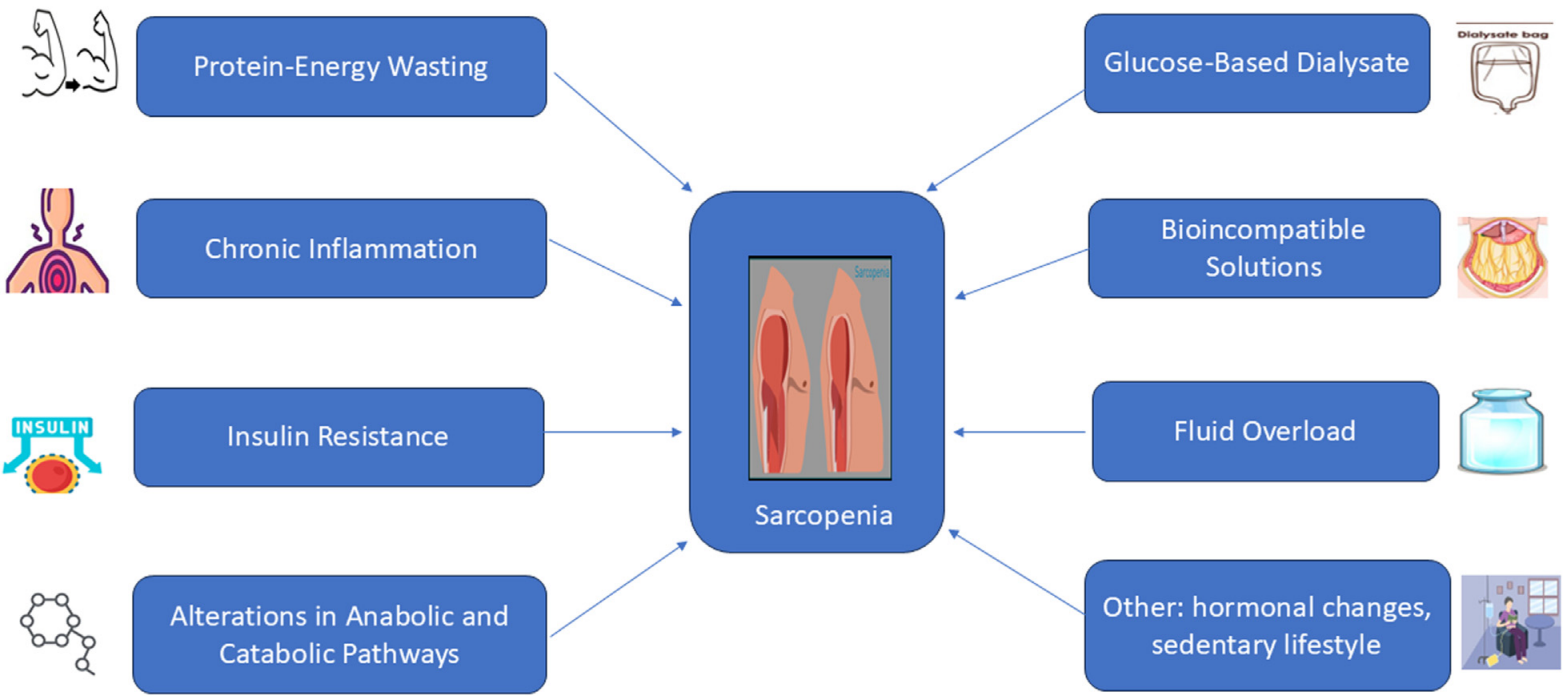
Mechanisms of sarcopenia in peritoneal dialysis.

#### Protein-Energy Wasting

The protein-energy wasting is a critical factor in the pathogenesis of sarcopenia among patients treated with PD.^14^ It is characterized by a decline in body protein mass and energy reserves, resulting from inadequate dietary intake, inflammation, and the catabolic effects of kidney disease itself. The regular loss of proteins through dialysis further exacerbates this condition. Westra et al^15^ demonstrated that automated PD incurs a loss of ∼10 g of protein per 24 hours, and was increased by dwell time and number of night time exchanges.

#### Chronic Inflammation

Patients treated with PD often experience a state of chronic inflammation, marked by elevated levels of pro-inflammatory cytokines such as tumor necrosis factor-α, interleukin-6, interleukin-1, and C-reactive protein. This inflammatory milieu contributes to muscle catabolism, inhibiting protein synthesis and promoting protein degradation, repercussions for the quality of life and long-term prognosis.^14^

#### Insulin Resistance

Insulin resistance is commonly observed in patients treated with PD and contributes to sarcopenia by impairing the anabolic effects of insulin on muscle, reducing muscle protein synthesis, and facilitating muscle protein breakdown.^12^

#### Alterations in Anabolic and Catabolic Pathways

The balance between muscle protein synthesis and degradation is disrupted in patients treated with PD. Factors such as increased myostatin levels, reduced growth hormone and insulin-like growth factor-1 signaling, and alterations in the testosterone and estrogen pathways contribute to reduced muscle mass and function.^12^

### PD-Specific Factors

The PD-specific factors included are as follows:

#### Glucose-Based Dialysate

The use of glucose-based dialysate solutions in PD can contribute to hyperglycemia and insulin resistance, further exacerbating muscle wasting. The high glucose content can also lead to increased fat deposition and reduced muscle protein synthesis. The exposure of patients treated with PD to glucose as an osmotic agent may lead to the absorption of up to 300 g of glucose per day, depending on the patient’s membrane profile and the prescription of hypertonic solutions. Such a glucose load has a direct impact on the patient’s appetite, reducing the daily intake of proteins and other nutrients.^16^

#### Bioincompatible Solutions

Chronic exposure to bioincompatible dialysis solutions can induce local and systemic inflammatory responses, leading to oxidative stress and endothelial dysfunction. These factors negatively affect muscle metabolism and can accelerate the progression of sarcopenia.^14^

#### Fluid Overload

Fluid overload, a common issue in patients treated with PD, can interfere with the accurate assessment of muscle mass and contribute to physical inactivity, further worsening sarcopenia.^17^

Moreover, hormonal changes associated with PD, such as reduced levels of anabolic hormones, exacerbate muscle loss. Lastly, the sedentary lifestyle often adopted by patients treated with PD due to fatigue and dialysis schedules compounds the risk of developing sarcopenia.^12^

Understanding the multifactorial pathophysiology of sarcopenia in patients treated with PD is crucial for developing targeted interventions. Addressing the underlying mechanisms through nutritional support, exercise, and potentially pharmacological interventions may help mitigate muscle wasting and improve outcomes for patients treated with PD.

### Diagnosis of Sarcopenia

Currently, there is no unified standard for the detection of sarcopenia, with the diagnosis based on assessments of muscle mass, muscle strength, and physical function. The most recent consensus definitions, such as those from EWGSOP2 and AWGS 2019, highlight that the diagnosis of sarcopenia requires not only the loss of muscle mass but also a decline in muscle strength or physical function (Table 1). These guidelines also introduce the concept of possible sarcopenia, shifting the diagnostic focus from solely low muscle mass to low muscle strength (hand grip strength—HGS) due to its ease of assessment and stronger predictive power for cardiovascular risks and mortality.

**Table 1:** Diagnostic Criteria of Sarcopenia

	Low Muscle Mass (ASM)	Low Muscle Strength	Lower Physical	Sarcopenia Diagnosis
		(HGS)	Performance (GS)	
AWGS (2019)	ASM/height^2^ < 7.0 kg/m^2^ for men and < 5.4 kg/m^2^ for women by using DXA ASM/height^2^ < 7.0 kg/m^2^ for men and < 5.7 kg/m^2^ for women by using BIA	HGS < 28 kg for men and < 18 kg for women	Usual gait speed < 1.0 m/s for both sexes	Possible sarcopenia: LMS or LPP Sarcopenia: LMM plus LMS or LPP Severe sarcopenia: LMM plus LMS and LPP
EWGSOP (2019)	ASM/height^2^ < 7.0 kg/m^2^ for men and < 5.5 kg/m^2^ for women	HGS < 27 kg for men and < 16 kg for women	Usual gait speed ≤0.8 m/s for both sexes	Possible sarcopenia: LMS Sarcopenia: LMM plus LMS Severe sarcopenia: LMM plus LMS and LPP

Abbreviations: ASM, appendicular skeletal muscle; AWGS, Asian Working Group for Sarcopenia; BIA, bioelectrical impedance analysis; DXA, dual-energy X-ray absorptiometry; EWGSOP, European working group on sarcopenia in older people; GS, gait speed; HGS, handgrip strength; LMM, lower muscle mass; LMS, lower muscle strength; LPP, lower physical performance.

The clinical diagnosis of sarcopenia involves a multifaceted approach, incorporating assessments of muscle mass, strength, and physical performance. The most widely accepted criteria for diagnosing sarcopenia include: (1) muscle mass measurement: techniques such as dual-energy X-ray absorptiometry and BIA are commonly used. The dual-energy X-ray absorptiometry provides a detailed body composition analysis, distinguishing between bone, fat, and lean muscle mass, making it a gold standard for muscle mass measurement. The BIA, on contrary, estimates body composition based on the electrical conductivity of various tissues, offering a noninvasive and quicker alternative, albeit with certain limitations regarding accuracy and consistency. (2) Physical performance tests: these include the gait speed test, timed up-and-go test, and the 6-minute walk test, which assess an individual’s mobility and endurance. A decline in performance in these tests indicates compromised physical function, a key component of sarcopenia diagnosis. (3) Strength assessments: HGS, measured using a dynamometer, is the most common test for muscle strength assessment. It is simple, quick, and has been strongly correlated with overall muscle strength and health outcomes.

The strength, assistance walking, rise from a chair, climb stairs, and falls (SARC-F), a 5-item self-reported questionnaire first developed in 2013,^18^ is a well-established and widely used initial screening tool for geriatric sarcopenia and has been recently recommended by AWGS 2019 and the revised EWGSOP2. One study has been reported use of SARC-F among patients undergoing PD. This study found that the SARC-F had a high negative predictive value and a high specificity for predicting sarcopenia in patients treated with PD.^19^ The recent report compared the diagnostic performance of SARC-F, SARC-F combined with calf circumference (SARC-CalF), and calf circumference (CC) for screening sarcopenia among patients undergoing PD found that CC and SARC-CalF outperformed SARC-F in the diagnostic accuracy of sarcopenia among patients undergoing PD.^20^ The clinical use of SARC-F, CC, and SARC-CalF among the PD need to be evaluated in further studies.

Diagnosing sarcopenia in patients treated with PD presents unique challenges, primarily due to the effect of fluid overload on the accuracy of muscle mass measurements: (1) fluid overload: patients treated with PD often experience fluctuations in hydration status, which can significantly affect the readings obtained from both dual-energy X-ray absorptiometry and BIA. Fluid overload can lead to overestimation of muscle mass, masking the true extent of sarcopenia. This necessitates careful interpretation of results and, potentially, the use of adjusted criteria or correction formulas to account for the fluid status. (2) Variability in clinical presentation: the presence of comorbid conditions, variations in residual renal function, and the effect of the dialysis process itself can all influence the clinical presentation of sarcopenia in patients treated with PD, complicating the diagnostic process. Addressing these challenges requires a comprehensive understanding of sarcopenia’s clinical manifestations and the limitations of current diagnostic tools in the context of PD. Future advancements in diagnostic methodologies and tailored criteria for patients treated with PD will be crucial in overcoming these obstacles, ensuring accurate diagnosis and effective management of sarcopenia in this vulnerable population.

For the screening and diagnosis of sarcopenia, EWGSOP2 recommends following the F-A-C-S (find cases—assess—confirm—severity) pathway (Table 2), involving various diagnostic tools, indicators, and thresholds. Although the AWGS and EWGSOP2 consensuses target the elderly, CKD is an independent factor affecting sarcopenia, irrespective of age. The applicability of existing diagnostic standards to CKD and patients treated with dialysis requires validation through further studies.

**Table 2:** For Screening and Diagnosis of Sarcopenia, EWGSOP2 Recommends Following the Pathway: Find Cases-Assess-Confirm-Severity (F-A-C-S)

	Cut-Off Points	Diagnosis
Find cases	SARC-F (simple 5-item questionnaire) scores ≥4 or clinical suspicion (ie falling, feeling weak, slow walking speed, difficulty rising from a chair, or weight loss/muscle wasting)	
Assess	Muscle strength Grip strength: men < 27 kg, women <16 kg Chair stand > 15 seconds for 5 rises	Sarcopenia probable^a^
Confirm	Muscle quantity or quality (measured by dual-energy X-ray absorptiometry or bioelectrical impedance analysis or magnetic resonance imaging or computed tomography) Appendicular muscle mass: men < 20 kg, women < 15 kg Appendicular muscle mass/height^2^: men < 7.0 kg/m^2^, women < 5.5 kg/m^2^	Sarcopenia confirmed
Severity	Physical performance Gait speed ≤ 0.8 m/s Short physical performance battery ≤ 8 score Timed-up-and-go test ≥ 20 seconds 400 m walk: non-completion or ≥ 6 minutes for completion	Sarcopenia severe

a Consider other reasons for low muscle strength (eg, depression, stroke, balance disorders, and peripheral vascular disorders).

### Predictive Markers for Sarcopenia

The diagnosis of sarcopenia necessitates using 3 different measurement tools to assess muscle mass, muscle strength, and physical function comprehensively. Although muscle mass testing may involve sophisticated imaging or bioelectrical impedance testing methods, grip strength, and gait speed testing could be influenced by physical conditions, making the screening process more complicated. Recent studies have suggested simplified diagnostic procedures and explored correlations between serological indicators and sarcopenia, indicating a need for further research on their predictive efficacy. New markers like phase angle (PhA) and irisin have emerged as promising tools for sarcopenia diagnosis, potentially offering more convenient options for clinical practice.

The PhA, derived from BIA, reflects cell membrane integrity and body cell mass. The BIA is a method of analyzing human body composition by introducing weak alternating current to obtain impedance values. The PhA is used as the main marker for detection. Some studies showed the association between PhA and mortality or malnutrition in patients undergoing PD. Fein et al showed a positive correlation between PhA and albumin, total protein, and creatinine in 45 patients undergoing PD.^21^ Mushnick et al. enrolled 48 patients undergoing PD and showed that PhA is associated with patient survival and serum albumin level.^22^ Low PhA is linked to increased mortality, serum albumin, creatinine, and good residual renal function in patients receiving PD, according to Huang et al large sample of 760 patients undergoing PD.^23^ In a recent report,^24^ they enrolled prevalent patients undergoing PD (n = 200). Patients were divided into tertiles based on PhA levels: low, middle, and high. Those in the low tertile had significantly higher odds of developing sarcopenia, with an odds ratio of 9.8 compared with the middle tertile and 52.79 compared with the high tertile. This study demonstrated that PhA was independently linked to muscle mass, strength, and sarcopenia in patients treated with PD, suggesting it could be a more practical diagnostic tool than current sarcopenia criteria.

Irisin, a myokine released during physical exercise, plays a crucial role in enhancing muscle mitochondrial function and counteracting myostatin formation.^25^ Lee et al^26^ showed that serum irisin was positively correlated with mid-arm muscle circumference and thigh circumference, which suggested that serum irisin was significantly associated with sarcopenia in PD.

The combination of serum irisin concentrations and PhA has shown promise in rapidly predicting sarcopenia among patients treated with PD, potentially serving as an optimal screening tool in clinical settings. Wu et al^27^ reported that the combination of serum irisin concentrations and PhA facilitated the rapid prediction of PD sarcopenia and could serve as an optimal screening tool for PD sarcopenia in clinical settings. A machine learning-based model^28^ incorporating these markers, along with other clinical features, has demonstrated effectiveness in predicting PD sarcopenia, highlighting the clinical potential of these markers as convenient tools for sarcopenia screening.

These emerging markers, PhA, and serum irisin, offer new avenues for the early detection and management of sarcopenia in patients treated with PD. By integrating these markers into clinical practice, health care providers can enhance the diagnosis and treatment of sarcopenia, ultimately improving patient outcomes and quality of life.

### Association With Clinical Outcomes

Patients who were diagnosed with sarcopenia by combined criteria had a higher risk of mortality than those without sarcopenia.^10^ Research has particularly focused on sarcopenia's predictive capability for mortality among patients treated with PD, exploring how muscle mass, strength, and sarcopenia, as composite diagnostic indicators, correlate with patient outcomes.

Kim et al^29^ analyzed 131 patients treated with PD, finding that low HGS presented a lesser predictive ability for mortality compared with changes in lean or fat tissue index. This underscores the complexity of sarcopenia's impact, suggesting that muscle strength may not solely determine mortality risk. Furthermore, various indices of muscle mass have been investigated, revealing a significant association with mortality in patients treated with PD.^30^^,^^31^ This highlights the crucial role of muscle health in influencing survival rates among this population.

Kang et al.'s study involving 199 patients treated with PD indicated that HGS might be a more accurate predictor of patient or technique survival than muscle mass or sarcopenia.^9^ This suggests that evaluating muscle strength could offer valuable insights into patient outcomes, potentially guiding interventions to enhance longevity and quality of life for those undergoing PD.

Cardiovascular disease (CVD) is a highly common complication and the first cause of death in patients undergoing dialysis. A meta-analysis demonstrated that sarcopenia was one of the most important predictors of CVD events and mortality outcomes in patients treated with HD.^7^ There are few studies on the relationship between sarcopenia and CVD events in patients treated with PD. In one study conducted on 129 patients treated with dialysis, the authors failed to find an association between sarcopenia and CVD events or all-cause mortality.^32^

The coexistence of sarcopenia and obesity, namely sarcopenic obesity, has garnered increasing attention due to its association with poorer survival in both general and elderly populations. Do et al^33^ retrospectively evaluated the association between sarcopenia and obesity from 199 patients treated with PD. After 18 months of follow-up, they reported that patients with sarcopenic obesity had a significantly lower survival rate compared with those with nonsarcopenic nonobesity. The present prospective study showed that sarcopenic obesity served as an independent predictor for higher mortality rates in patients treated with PD, which was following previous studies.^34^

Kamijo et al. analyzed the association of sarcopenia with frailty based on the clinical frailty scale (CFS). This study found that sarcopenia was significantly correlated with frailty.^10^ A study reported that enrolled 368 patients treated with PD using the CFS, 19.3% of patients were classified as frail, compared with 17.7% with sarcopenia. The presence of sarcopenia or frailty was associated with a worse prognosis in patients treated with PD.^35^

Sarcopenia has a poor prognosis for patients with PD. However, the current discourse on the prognostic value of muscle mass versus muscle strength in patients treated with PD with sarcopenia remains contentious. Future research is needed to discern the most effective survival indicator, aiming to refine diagnostic criteria and therapeutic approaches for managing sarcopenia in patients treated with PD.

### Interventions of Sarcopenia in PD

Interventional strategies for sarcopenia, especially within the context of PD (Fig 3), focus primarily on mitigating the loss of muscle mass and strength through nutritional supplementation and physical exercise. The summary treatment effect on each parameter of sarcopenia, such as muscle mass, muscle strength, or physical performance (Table 3).^36^, ^37^, ^38^, ^39^, ^40^, ^41^, ^42^, ^43^ Research in this area, though limited, underscores the critical role of protein supplementation and resistance training in managing sarcopenia among patients treated with PD.

**Figure 3:**
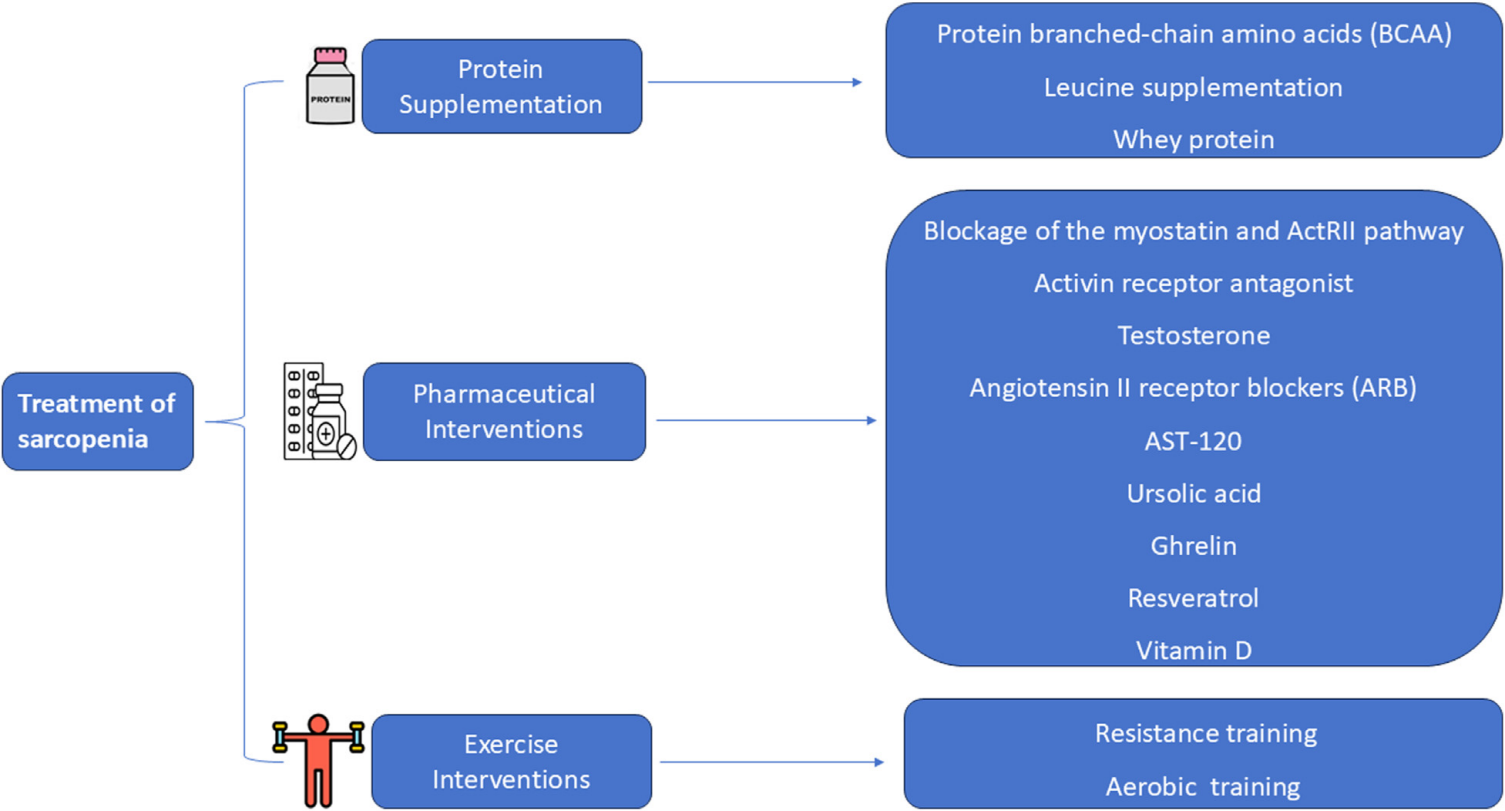
Treatment of sarcopenia in peritoneal dialysis.

**Table 3:** The Summary Treatment Effect on Each Parameter of Sarcopenia, Such as Muscle Mass, Muscle Strength, or Physical Performance

Study	Country	Dialysis Modality	Sample Size	Type of Intervention	Length of Follow-Up	Sarcopenia Related Outcomes
González-Espinoza et al^36^ (2005)	Mexico	PD	28	Oral egg albumin-based supplement	6 mo	MAMA
Sahathevan et al^37^ (2018)	Malaysia	PD	74	Whey protein supplementation	6 mo	HGS MAMA MAMC
Luo et al^38^ (2020)	China	PD	142	Nurse-led personalized dietary plans	12 mo	MAMC
Johansen et al^39^ (1999)	United States	HD and PD	29	Nandrolone	6 mo	LBM
Uchiyama et al^40^ (2019)	Japan	PD	47	Aerobic and resistance training	12 wk	HGS
Bennett et al^41^ (2020)	United States	PD	36	Aerobic and resistance training	12 wk	TUG
Manfredini et al^42^ (2017)	Italy	HD and PD	296	Walking exercise	6 mo	STS
Molsted S et al^43^ (2013)	Denmark	HD and PD	29	High-load strength training and protein intake	48 wk	Knee extension STS

Abbreviations: PD, peritoneal dialysis; MAMA, mid-arm muscle area; HGS, handgrip strength; MAMC, mid-arm muscle circumference; HD, hemodialysis; LBM, lean body mass; TUG, timed-up-and-go; STS, sit-to-stand.

#### Protein Supplementation

Chronic kidney disease and kidney failure are characterized by abnormalities in amino acid metabolism, notably involving branched-chain amino acids and their keto acids (BCKA).^44^ Leucine supplementation, in particular, has been shown to enhance muscle protein synthesis in older adults.^45^ Whey protein emerges as a highly recommended nutritional supplement for sarcopenia, as advocated by the AWGS 2019 consensus. The 2020 updated practice guideline for nutrition in CKD from KDOQI-NKF guidelines recommends a protein intake of 1.0-1.2 g/kg/day for patients with PD.^46^ Two trials reported that protein supplementation in patients treated with PD improved nutritional condition, but with no significant difference in muscle mass or muscle strength.^36^^,^^37^ One study showed that nurse-led food exchange model intervention improved the nutrition condition and middle-arm muscle circumference of patients treated with PD dramatically.^38^ This approach is supported by evidence suggesting that dietary protein intake plays a pivotal role in the treatment of sarcopenia, helping to counterbalance the protein losses through PD and stimulate muscle protein synthesis.

#### Pharmaceutical Interventions

Targeting specific pathways involved in muscle catabolism and inflammation could offer therapeutic avenues for sarcopenia. Myostatin, a negative regulator of muscle growth, has been identified as a potential target. Elevated inflammatory states were associated with kidney failure, myostatin inhibitors could theoretically promote muscle hypertrophy and counteract muscle loss.^47^ Different study results have indicated that blocking the myostatin and ActRII pathways had a significant effect on muscle hypertrophy. However, no significant effect on muscle strength or physical function has been observed.^4^ Johansen et al^39^ reported that a 6-month treatment with nandrolone, an anabolic steroid, led to a notable increase in lean body mass and was linked to functional improvements in patients undergoing dialysis. Other therapeutic strategies being studied or in trial phases for the treatment of sarcopenia include activin receptor antagonists, follistatin fusion proteins, gene therapy, testosterone, Angiotensin II receptor blockers, resveratrol, ghrelin, vitamin D, ursolic acid, and oral spherical carbon adsorbent (AST-120).^4^^,^^48^ However, despite promising results in experimental settings, pharmaceutical interventions remain underexplored in clinical practice, except for specific scenarios such as amino acid or keto acid analog supplementation.^4^

#### Exercise Interventions

Physical activity, particularly resistance training, has been identified as the most potent intervention for sarcopenia in dialysis patients. The recommendations of the International Society for Peritoneal Dialysis and the Global Renal Exercise Network, emphasize the importance of integrating physical activity into the routine care of patients treated with PD.^49^ However, nephrologists and nephrology nurses frequently lack the knowledge, resources, and skills to prescribe detailed or appropriate exercise regimens. A randomized controlled trial in patients treated with PD found no changes in handgrip strength but significant improvements in physical role functioning following a 12-week home-based exercise program.^40^ Another study found that the resistance and cardiovascular exercise program appears feasible and safe for patients treated with PD. They recommended that providers of PD therapy consider including exercise programs coordinated by exercise professionals to reduce the physical deterioration of patients treated with PD.^41^ Manfredini et al^42^ reported that walking exercise for 6 months could improve physical performance and quality of life in patients treated with dialysis. Moreover, high-load strength training was associated with improvements in muscle strength and power, physical performance, and quality of life with dialysis.^43^ Although evidence supporting the efficacy of exercise interventions in the population treated with PD is limited, intradialytic exercises have shown promise in modifying sarcopenia measures in patients treated with HD, indicating potential benefits for patients treated with PD as well.^50^

Combining oral energy and protein supplementation with supervised physical resistance exercise represents a comprehensive approach to reversing sarcopenia in patients treated with PD. Oikawa et al study highlights the benefits of combining whey protein intake with resistance exercise, showing significant improvements in muscle synthesis and overall muscle health.^51^ A 24-week randomized controlled trial in elderly women further corroborated the superiority of combined interventions over single interventions in improving muscle mass, grip strength, and physical function, although the long-term benefits remain to be fully determined.^52^

Although the research on interventions for sarcopenia in PD is still evolving, current evidence points to the effectiveness of protein supplementation and exercise in improving muscle health among patients treated with PD. Future studies are needed to expand our understanding of these interventions and explore new therapeutic targets for sarcopenia in this population.

## Conclusions

In summary, sarcopenia is a prevalent and consequential complication in patients undergoing PD, significantly impacting mortality and quality of life. Despite the lack of standardized criteria for the diagnosis of sarcopenia in this population, routine screening using available methods is essential for early detection and intervention. The complexity of sarcopenia, characterized by muscle mass loss, decreased strength, and diminished physical performance, necessitates a multidimensional approach to diagnosis and management.

Current interventions for sarcopenia, particularly in patients treated with PD, are limited in number but highlight the importance of oral energy and protein supplementation combined with physical activity. Emerging research on predictive markers and models for sarcopenia offers promising directions for simplified diagnostic procedures and targeted interventions. However, the development of reliable diagnostic methods specific to patients treated with PD remains a challenge, underscoring the need for further research in this area.

Effective management of sarcopenia in patients treated with PD requires an integrated approach that addresses the multifactorial etiology of the condition. This includes optimizing nutritional status, encouraging regular physical exercise, and potentially exploring pharmaceutical interventions targeting muscle metabolism and inflammation. As the field advances, it is hoped that new insights and therapeutic strategies will emerge to improve the care and outcomes of patients treated with PD with sarcopenia.

This review underscores the critical need for heightened awareness and proactive management of sarcopenia in patients treated with PD. By advancing our understanding of the condition’s etiology, diagnostic criteria, and effective interventions, health care providers can better support the health and well-being of this vulnerable population.
